# Locomotive syndrome in cancer patients: a new role of orthopaedic surgeons as a part of comprehensive cancer care

**DOI:** 10.1007/s10147-022-02194-w

**Published:** 2022-06-11

**Authors:** Hirotaka Kawano, Masahiro Hirahata, Jungo Imanishi

**Affiliations:** Department of Orthopaedic Surgery, Teikyo University School of Medicine, 2-11-1, Kaga, Itabashi-ku, Tokyo, 173-8605 Japan

## We have entered the cancer era

In 2016, the number of new cancer cases per year in Japan finally exceeded one million [1]. Shockingly, the number also far exceeded the number of new births. Because the prognosis of cancer patients has improved in the last few decades, thanks to advances in cancer treatment, we are having an increasing number of cancer patients at any stage, including those with bone metastases [2]. As the world’s first super-aged society, Japan has already entered the “cancer era”, and is experiencing a paradigm shift in cancer care where not only complete cure of cancer but also care of cancer patients aiming to maintain and improve their quality of life (QOL) is required. It may be needed to accept cancer as a chronic disease and to aim to maintain and improve QOL of cancer patients [3].

## “The curse of cancer treatment” hinders comprehensive approach for cancer

It seems customary that both of medical providers and cancer patients prioritize cancer over other diseases. Behind this custom, it is felt that as if there were the "curse of cancer treatment" that coerces the thought that cancer must be cured completely. In Japan, “cancer” is often used as a metaphor for a person who has a bad effect on all the others in a group. This trend is not necessarily unique to Japan but rather can be universal. Vrinten et al. conducted a systematic review and meta-synthesis of “cancer fear” in cancer screening, using thematic synthesis [4]. The authors analyzed 102 relevant studies and labelled cancer as the enemy. They interpreted that fears of cancer emanated from a view of cancer as a vicious, unpredictable, and indestructible “enemy”. Such a view, regarding cancer as the enemy, can force us to fight against cancer. This fight may also narrow the field of view, and compel both of medical providers and patients to pursue the prognosis of life only. As a result, only direct cancer treatment, such as surgery, chemotherapy, and radiotherapy, has tended to be focused on for cancer patients, sometimes until the end of life [5]. Even the provision of palliative care, which is an essential part of cancer care, has been limited [6]. The establishment of comprehensive cancer care, by implementing missing elements and optimizing the balance of biomedicine and the soul of medicine, is warranted [7].

## Do not orthopaedic surgeons see cancer patients?

Orthopaedic surgeons may have been at the very center of another “curse of cancer treatment”. They deal mainly with trauma and degenerative diseases, thus most of them are indifferent to cancer and have limited opportunities to participate in cancer treatment. Although the sub-specialty of bone and soft tissue tumor exists, its main target has been bone and soft tissue sarcoma. Because “sarcoma” is a rare cancer, the number of orthopaedic surgeons specializing in bone and soft tissue tumor is also limited; the number of Japanese board-certified orthopaedic surgeons specializing in bone and soft tissue tumor was only 161 in April 2022, whereas that of the whole certified orthopaedic surgeons by the Japanese Orthopaedic Association (JOA) was 20,954 [8]. Sarcoma has been thought to be the disease that only specialists should deal with, and orthopaedic surgeons have been repeatedly educated to refer patients on suspicious of sarcoma to specialists immediately. This awareness-raising activities about sarcoma has been successful, and the frequency of unplanned excision of sarcoma by orthopaedic surgeons has been reduced [9]. However, they may have misunderstandingly extended “Do not touch malignant tumor” principle into all cancers.

In 2018, JOA conducted a questionnaire survey to investigate the current involvement of orthopaedic department in cancer management at the orthopaedic training facilities certified by JOA. In the survey, more than a half of the designated cancer hospitals answered that they were not involved in cancer treatment including bone metastasis. Approximately 80% of the respondents from facilities other than designated cancer hospitals answered that they had no plans to be involved in the future [10].

There must be many cancer patients at the designated cancer hospitals nationwide. On the other hand, there are only a limited number of hospitals that have bone and soft tissue oncologists. Orthopaedic surgeons tend to avoid being involved when confronting cancer patients, as they think they are inexpert. As a result, cancer patients with locomotive dysfunction often miss the opportunity to receive appropriate treatment only because they are cancer patients. It should be noted that the average age of cancer patients is 75 years [1], and older people have a higher frequency of locomotive dysfunction, such as spondylosis deformans and osteoarthritis, before they develop cancer [11]. Restrictions on activities of daily living (ADL) due to locomotive dysfunction can also affect performance status (PS) and cause discontinuation of cancer treatment because poorer PS is generally a contraindication of chemotherapy for cancer.

## Performance status and locomotive dysfunction

PS is a worldwide measure of a patient’s daily living abilities, widely used in oncology research and practice [12]. PS 0 is defined as “fully active, able to carry on all pre-disease performance without restriction”, whereas PS 4 as “completely disabled; cannot carry on any self-care; totally confined to bed or chair”. PS 2, “ambulatory and capable of all self-care but unable to carry out any work activities; up and about more than 50% of waking hours”, is often a borderline of chemotherapy indication. It is certain that PS is largely affected by locomotive dysfunction, but caution must be taken when poor PS due to locomotive dysfunction is caused by reversible factors.

PS is defined as the degree of ADL restriction due to “cancer”, and the restriction due to temporary locomotive dysfunction should not be applied [12]. For example, if a cancer patient who was fully active has a femoral neck fracture, apparent PS may become 3–4, but it is expected to improve after surgical intervention. As in this example, apparent poor PS should not be regarded as a contraindication of cancer treatment if it is reversible. Certainly, orthopaedic surgeons have potential to improve apparent poor PS due to locomotive dysfunction, and further broaden the therapeutic indications for cancer.

## Locomotive syndrome in cancer patients (Cancer Locomo)

In 2007, JOA proposed the concept of “locomotive syndrome”, which is a condition of reduced mobility due to impairment of locomotive organs [13] as an educational activity in the orthopaedic field, and has promoted the campaign of “prevention of locomotive syndrome”. The awareness of locomotive syndrome has been increasing thanks to educational activities, and the concept that the prevention of locomotive syndrome is crucial for longer and healthier life has become widely recognized. However, there still remains an area where such activities can make another significant contribution. That is exactly “cancer care”.

One in two people in Japan is currently affected by cancer in their lifetime [1]. In the cancer era of the super-aged society, the need of management of locomotive dysfunction, especially in cancer patients, is increasing. It appears that orthopaedic surgeons are called to change their attitudes towards cancer. In response to such a trend demanding more involvement of orthpaedic surgeons in cancer treatment, JOA decided the activity theme as “locomotive syndrome in cancer patients” in 2018.

**Fig. 1:**
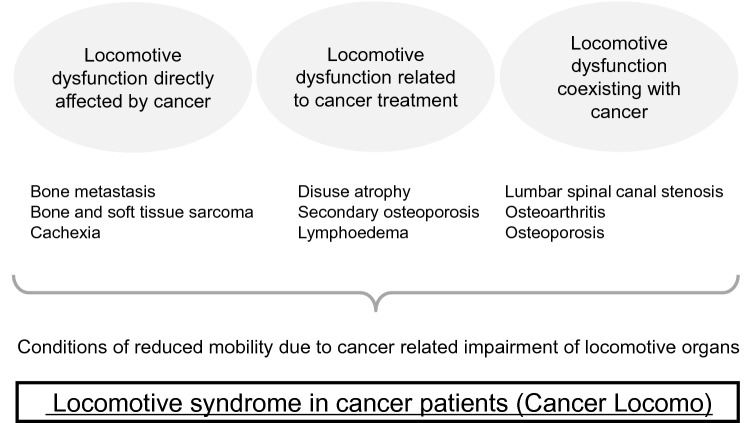
Three types of locomotive syndrome in cancer patients

Locomotive syndrome in cancer patients is defined as “a condition of reduced mobility due to cancer-related impairment of locomotive organs”, and can be classified into three types (Fig. 1). Type 3 may look remotely related to cancer, but this type is clinically the most important; this type can be neglected or deprioritized by orthopaedic surgeons who are indifferent to cancer.

1. Locomotive dysfunction directly affected by cancer
  This type includes the problems of the locomotor system arising from cancer itself (e.g., bone metastasis, bone and soft tissue sarcoma, and cachexia).
2. Locomotive dysfunction related to cancer treatment
  This type includes disuse atrophy due to long-term bed rest, secondary osteoporosis, peripheral neuropathy, lymphoedema and joint contracture after cancer treatment. Osteoporosis can be caused by steroid use and hormone therapy. Peripheral neuropathy can be caused by chemotherapy. Lymphoedema and joint contracture can be caused by radiotherapy or surgery.
3. Locomotive dysfunction coexisting with cancer
  This type includes problems of the general locomotor diseases, such as osteoporosis, lumbar spinal canal stenosis and osteoarthritis. The problems are sometimes neglected or deprioritized by orthopaedic surgeons simply because of the existence of “cancer”.

Little has been known on locomotive syndrome in cancer patients hitherto because this concept is new and only a few studies have been published. The lack of information is partially because of the indifference of orthopaedic surgeons towards cancer. Systematic review on clinical results of orthopaedic surgery for cancer patients was once conducted, but virtually no publication was found. The majority of orthopaedic clinical studies have excluded "cancer patients" from their subjects [14–16].

JOA has started "Survey on the actual condition of locomotive syndrome in cancer patients" as project research of the society. Although limited data are available at this time, Sato et al. have recently revealed that incidence of locomotive syndrome in cancer patients was much higher than that of the general population, 51% vs. 13% for LS stage 2 [17].

The campaign of “Locomotive syndrome in cancer patients" is the activity where not only orthopaedic oncologists but all orthopaedic surgeons, are expected to actively participate in cancer care. Through this activity, even if the cancer itself is incurable, the cancer patients are expected to receive appropriate locomotor management and maintain their independent life as long as possible. Orthopaedic surgeons can greatly contribute to cancer care by improving the QOL of cancer patients, using their professional expertise.

Looking at the situation in other medical care areas, new fields associated with cancer, such as onco-cardiology and onco-nephrology [18, 19], have been already established. Both address problems specific to cancer patients. Similarly, a new field can be created for orthopaedic problems specific to cancer patients, namely locomotive disorders peculiar to cancer patients or locomotive syndrome in cancer patients. This new field, the management of locomotive syndrome in cancer patients, may be called “onco-orthopaedics” following the precedents.

## Ideal care for bone metastasis

Bone metastasis is a major cause of locomotive syndrome in cancer patients. Although the actual prevalence of bone metastasis of each cancer is difficult to know, it is said that bone metastasis is present in more than a half of the cancer patients at the terminal stage, and 20% of the cancer patients at terminal stage have clinical symptoms [20]. As the advance in cancer treatment has prolonged the overall survival of cancer patients, the survival time of patients with advanced cancer has also been extended [21].

For a long period of time, it may have been recognized that bone metastases occur near the end of life, and a fundamental or essential approach towards bone metastases have been seldom taken by primary cancer doctors and orthopaedic surgeons. When a diagnosis of bone metastasis is made radiologically, an incurable "stage IV" is often declared by the primary cancer doctors and palliative medicine or supportive care becomes the main focus. There may have been many facilities where the treatment of bone metastases has been managed by the primary cancer doctors, radiologists, and palliative care doctors without the involvement of orthopaedic surgeons. However, it should be noted that the involvement of orthopaedic surgeons can dramatically improve the accuracy of assessments. The judgement of whether the symptoms are due to bone metastases or not will become more accurate with the involvement of orthopaedic surgeons. In addition, more appropriate clinical decision will be made on bone metastases, especially for femoral and spinal metastases at risk of pathological fractures and spinal cord paralysis by balancing risks and benefits of surgical intervention.

Bone metastasis treatment demands multi-disciplinary approach, and the involvement of many clinical departments is indispensable. In addition to medical doctors, other medical specialists, such as nurses, pharmacists, physiotherapists, occupational therapists, and medical social workers, are required to comprehensively work on the team medical care [22].

## Cooperation between cancer rehabilitation and the campaign of “locomotive syndrome in cancer patients”

Cancer rehabilitation is a medical care system for cancer patients approved in the health insurance system in Japan in 2010. It has been introduced at cancer designated hospitals and has achieved some clinical results including a reduction in the frequency of perioperative complications [23]. According to the homepage of the National Cancer Center in Japan, cancer rehabilitation is defined that "improves the resilience of cancer patients, maintains and improves their remaining abilities." In contrast, the campaign of locomotive syndrome in cancer patients can be said to be the activity that "supports the maintenance and improvement of the mobility of cancer patients through locomotive management." For example, when a femoral pathological fracture due to bone metastasis occurs, in cancer rehabilitation, ADL training such as wheelchair transfer utilizing the remaining function is the choice on the premise of inability to walk due to the pathological fracture. However, in the concept of Cancer Locomo, walking ability itself can be restored by performing internal fixation or artificial joint replacement. Under cooperation between “cancer rehabilitation” that comprehensively manages cancer patients and “Cancer Locomo” that emphasizes mobility, the ADL and QOL of cancer patients can be maximized more than ever.

## Significance of “ambulatory ability” in cancer patients

Now that the period of living with cancer has been extended, ambulatory ability is essential for cancer patients to spend their own independent lives until the end of life, to keep working, and to continue cancer treatment. Through this campaign of locomotive syndrome in cancer patients, we would like all cancer careers to know that it is very effective to manage the locomotor function and maintain ambulatory ability. Although it is mandatory to further clarify the actual conditions of locomotive syndrome in cancer patients and verify the intervention effects of locomotive management, Orthopaedic surgeons, who have been far from "cancer", can and should contribute as an essential team member of cancer care using their professional expertise and skills.
